# Tropomyosin 3 Gene Fusions in Cancers: From Mechanisms to Treatments—A Comprehensive Review

**DOI:** 10.1002/cam4.71407

**Published:** 2025-11-23

**Authors:** Anjie Chen, Sixin Li, Chen Guo, Chenwei Gu, Jiandong Gui, Yujie Deng, Xichen Feng, Yuanyuan Mi

**Affiliations:** ^1^ Department of Urology Affiliated Hospital of Jiangnan University Wuxi Jiangsu Province China; ^2^ Wuxi School of Medicine Jiangnan University Wuxi Jiangsu Province China

**Keywords:** gene fusion, oncogenesis, therapeutic targeting, tropomyosin 3, tumor progression

## Abstract

**Background:**

Tropomyosin 3 (TPM3), one of the four tropomyosin genes, is predominantly expressed in eukaryotic cells. As a crucial regulatory protein, TPM3 associates with actin within thin myofilaments, thereby playing an essential role in the regulation of muscle contraction. Beyond its fundamental function in muscle physiology, TPM3 is implicated in oncogenesis.

**Objective:**

This review elucidates the molecular mechanisms underpinning TPM3 gene fusions, delineates the tumor types associated with these fusions, and examines their clinical implications.

**Findings:**

Gene fusions such as TPM3—NTRK1, TPM3—ALK, and TPM3—ROS1 have been identified as oncogenic drivers in various cancers. These fusions promote constitutive activation of tyrosine kinases, disrupt normal cellular signaling, and consequently accelerate tumorigenesis. Malignancies harboring TPM3 fusions encompass several tumor categories. With the advent of tyrosine kinase inhibitors (TKIs) targeting NTRK1, ALK, and ROS1 fusions, these rearrangements have gained significant therapeutic relevance. However, resistance mechanisms and tumor heterogeneity pose ongoing challenges to targeted therapy.

**Conclusion:**

By synthesizing current evidence, this review aims to provide insights into the diagnostic, prognostic, and therapeutic landscape of TPM3—related gene fusions, fostering advancements in precision oncology.

## 1 Introduction

Gene fusions constitute pivotal genomic alterations resulting from chromosomal rearrangements, which lead to the formation of hybrid genes [1]. These hybrid genes encode fusion proteins capable of disrupting normal cellular processes and contributing to oncogenesis. Of particular interest, tropomyosin 3 (TPM3) gene fusions have been identified as significant contributors to tumorigenesis. The TPM3 gene, located on chromosome 1q22‐23, encodes a protein essential for stabilizing actin filaments and maintaining cytoskeletal integrity [2, 3, 4]. However, when TPM3 undergoes fusion with tyrosine kinase genes such as neurotrophic receptor tyrosine kinase 1 (NTRK1), anaplastic lymphoma kinase (ALK) or c‐ros oncogene 1 (ROS1), it acquires oncogenic potential by constitutively activating kinase signaling pathways. These signaling cascades, notably the MAPK/ERK, PI3K/AKT, and JAK/STAT pathways, promote unregulated cell proliferation, survival, and metastatic potential [5, 6].

TPM3 fusions, although infrequent, have been identified in a wide array of malignancies including colorectal cancer, non‐small cell lung cancer (NSCLC) [7, 8, 9], inflammatory myofibroblastic tumors (IMTs) [10, 11], and thyroid carcinomas [8, 12]. Despite their rarity, these fusions have garnered significant attention in oncology due to their potential as actionable targets for tyrosine kinase inhibitors (TKIs) agents such as larotrectinib, entrectinib, and crizotinib have shown substantial efficacy in tumors with TPM3 fusions, underscoring the promise of precision oncology. Nonetheless, considerable challenges persist including resistance to targeted therapies, diagnostic challenges in detecting rare fusions, and tumor heterogeneity.

This review examines the molecular mechanisms underlying TPM3 fusions, the associated tumor types, and the therapeutic implications of targeting these alterations. By synthesizing current evidence, we aim to provide insights into the evolving landscape of TPM3‐related malignancies, emphasizing the necessity for ongoing research to overcome therapeutic resistance and enhance clinical outcomes. Importantly, this work represents the first comprehensive effort to integrate findings across all reported TPM3 fusion partners and cancer types, with the goal of establishing a unified biological and clinical paradigm that has been lacking in the literature.

## 2 Molecular Mechanisms of TPM3 Gene Fusions

### 2.1 TPM3‐ALK Fusion

In the TPM3‐ALK fusion, the TPM3 gene fuses with the ALK gene, producing a chimeric protein characterized by constitutive ALK kinase activity. This aberrant activation triggers oncogenic signaling pathways, notably the JAK/STAT and RAS/MAPK pathways, thereby promoting malignant transformation. Furthermore, the TPM3‐ALK fusion involves the dimerization of the fusion protein, a process facilitated by the coiled‐coil domain of TPM3. This dimerization is essential for the activation of the ALK kinase domain, which subsequently phosphorylates downstream signaling molecules such as ERK1/2 and STAT3, resulting in enhanced cellular proliferation and survival [13].

The TPM3‐ALK fusion has been associated with a range of malignancies including IMTs, renal cell carcinoma (RCC), and other neoplasms. This fusion also contributes to the elucidation of resistance mechanisms to anaplastic lymphoma kinase (ALK) inhibitors. In certain instances, resistance to first‐generation ALK inhibitors, such as crizotinib, has been documented, thereby necessitating the development of second‐ and third‐generation inhibitors to circumvent resistance and enhance patient outcomes [14, 15, 16]. This ongoing research into the molecular mechanisms of TPM3‐ALK fusions and their role in cancer pathogenesis continues to provide valuable insights into potential therapeutic strategies and the development of more effective treatments for patients with ALK‐rearranged tumors [17]. Clinically, TPM3–ALK occurs across multiple histologies. Disease‐specific evidence and representative cases are consolidated in Sections 3.2 (IMT) and 3.3 (RCC).

### 2.2 TPM3‐NTRK1 Fusion

The TPM3‐NTRK1 fusion juxtaposes the 5′ region of TPM3 with the 3′ region of NTRK1, generating an oncogenic chimeric protein that encodes a constitutively active TRKA receptor. This rearrangement drives ligand‐independent dimerization mediated by the coiled‐coil domain of TPM3, resulting in autophosphorylation and aberrant activation of downstream signaling cascades including MAPK/ERK and PI3K/AKT pathways. These events collectively promote tumorigenesis by enhancing cell proliferation and survival [13].

Clinically, TPM3‐NTRK1 fusions represent recurrent molecular alterations identified across diverse malignancies, notably colorectal carcinoma and soft tissue sarcomas. In colorectal carcinoma, the TPM3‐NTRK1 rearrangement has been identified as a recurring event, although it occurs at a low frequency. The presence of this fusion in colorectal cancer cells, such as the KM12 cell line, has been associated with hypersensitivity to TRKA kinase inhibitors. This suggests that patients with tumors harboring the TPM3‐NTRK1 fusion may benefit from targeted therapies that inhibit TRKA kinase activity [18].

Moreover, the TPM3‐NTRK1 fusion is not limited to colorectal carcinoma but has also been observed in other tumor types including certain soft tissue sarcomas. These sarcomas often exhibit a myopericytic or haemangiopericytic growth pattern and may present in both pediatric and adult patients. The recognition of this fusion in various neoplasms highlights the importance of molecular diagnostics in identifying actionable targets for therapy, particularly with the advent of TRK inhibitors that have shown efficacy in treating tumors with NTRK gene fusions [19].

The dimerization‐dependent constitutive activation of TRKA underpins the fusion's transformative potential, establishing it as a critical therapeutic target for TRK inhibitors in precision oncology.

### 2.3 TPM3‐ROS1 Fusion

TPM3‐ROS1 has been identified as a significant oncogenic driver in various tumors. The TPM3‐ROS1 fusion gene encodes a constitutively active ROS1 kinase, leading to continuous activation of signaling cascades that enhance tumorigenesis. This fusion has been implicated in various cancers, notably NSCLC. In pediatric gliomas and glioneuronal tumors, the ROS1 fusion, resulting from microdeletions at 6q22, has been identified as an oncogenic driver, highlighting the significance of ROS1 fusions in pediatric tumors [20].

The TPM3‐ROS1 fusion is also implicated in IMTs, where it acts as a key oncogenic mechanism. IMTs are mesenchymal tumors that can arise in various body parts, and the presence of ROS1 fusions in these tumors suggests a potential target for TKIs, which could provide therapeutic benefits [15].

**FIGURE 1:**
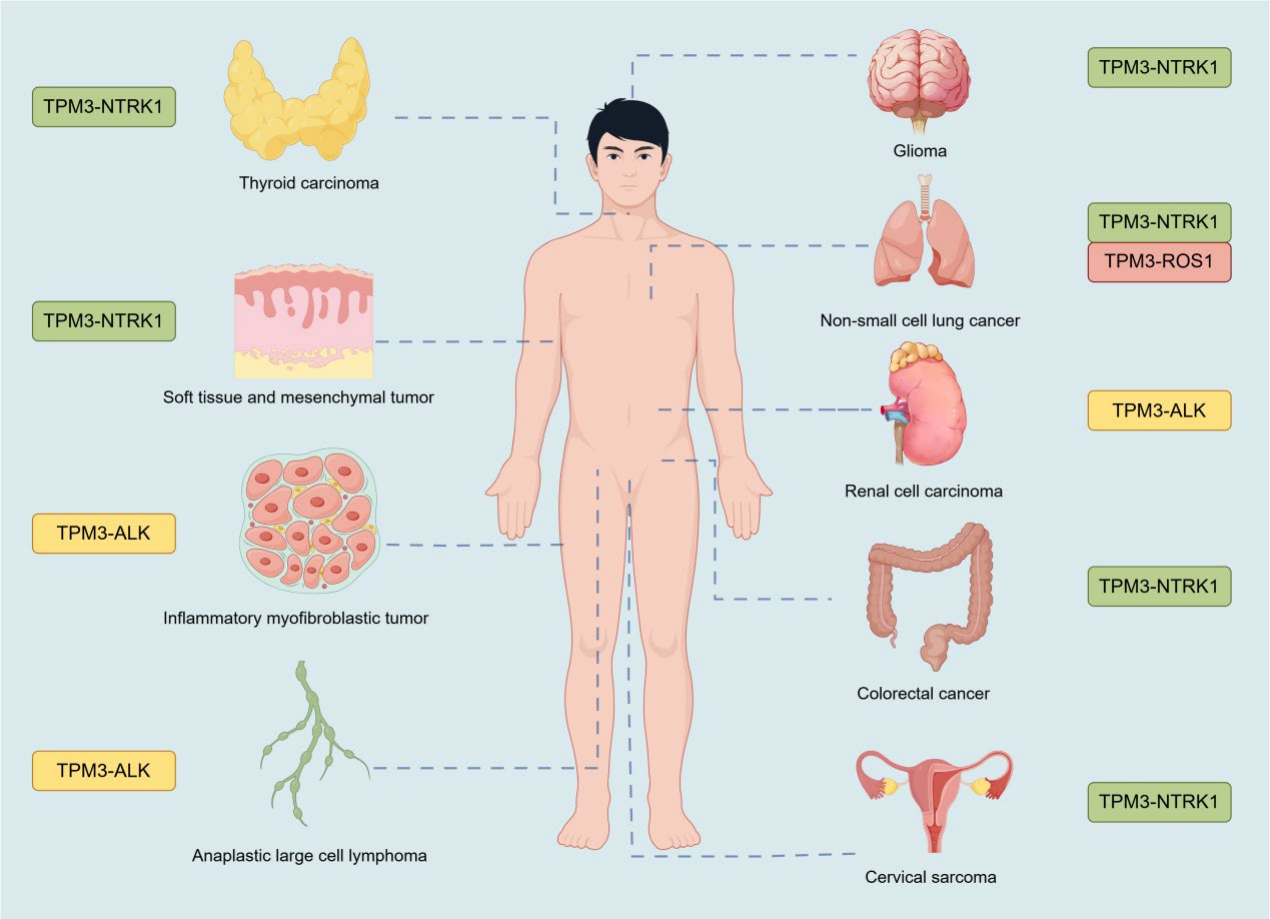
Spatial distribution of TPM3 gene fusions in human tumors. Schematic map showing tumor locations and their associated TPM3 fusion partners with color‐coded labels. Created with *Figdraw* (https://www.figdraw.com).

The oncogenic potential of TPM3‐ROS1 is further supported by its role in spindle cell carcinoma (SpCC) of the lung, where targeted therapy with crizotinib, a ROS1 inhibitor, has shown clinical efficacy. Moreover, the coexistence of TPM3‐ROS1 fusion with other gene fusions, such as EML4‐ALK, has been reported, indicating the complexity and heterogeneity of genetic alterations in cancer. This dual fusion scenario underscores the necessity for comprehensive molecular profiling to guide targeted therapy decisions [14]. For details, see Figure 1.

## 3 TPM3‐ALK Fusion‐Positive Tumors

### 3.1 Anaplastic Large Cell Lymphoma

Lamant et al. reported the TPM3‐ALK fusion gene in anaplastic large cell lymphoma (ALCL), emphasizing its role in activating the ALK protein. Their study highlighted how this fusion results in aberrant ALK expression, which is closely associated with the malignant transformation of lymphoid cells. This mechanism may explain the aggressive clinical course observed in ALCL patients [21]. In a similar context, Drexler et al. reviewed various ALK fusion partners including TPM3‐ALK, and examined its role in ALCL. They emphasized the importance of TPM3‐ALK as a fusion oncogene, not only in ALCL but also in IMTs. This work provided further support for TPM3‐ALK's involvement in these neoplastic processes [22]. Armstrong et al. described how TPM3‐ALK expression induces significant changes in cytoskeletal organization, which enhances metastatic capacity compared to other ALK fusion proteins. Their study demonstrated that TPM3‐ALK alters cell morphology and increases metastatic potential—both of which are critical factors in the progression of ALCL. Further extending this research, Giuriato et al. developed a conditional bioluminescent transplant model in 2007 to study TPM3‐ALK‐induced tumors, particularly fibroblastic tumors. Their results showed that TPM3‐ALK fusion protein is essential for tumor growth and maintenance. Moreover, they evaluated the effects of ALK TKIs and demonstrated that these inhibitors effectively hinder tumor progression [23]. In a follow‐up study in 2010, Giuriato et al. [24] used a mice model to further explore the oncogenic potential of TPM3‐ALK, underscoring the therapeutic potential of targeting ALK with specific inhibitors, showing notable tumor regression when treated with PF‐2341066, an ALK inhibitor. Melchers et al. reported a case of primary cutaneous ALCL driven by the TPM3‐ALK fusion, which was identified through RNA sequencing. This sequencing revealed a translocation t(1;2)(q25;p23) between the TPM3 and ALK genes, confirming TPM3‐ALK as an oncogene involved in the pathogenesis of cutaneous ALCL [25]. Luedersen et al. explored various ALK fusion variants in ALCL and highlighted TPM3‐ALK as one of the most prevalent. They noted that TPM3‐ALK exhibits higher metastatic potential in vitro compared to other ALK fusion variants such as ATIC‐ALK. Additionally, their study suggested that the presence of TPM3‐ALK in ALK‐positive ALCL is associated with a more favorable prognosis, as patients with TPM3‐ALK‐positive ALCL show a lower relapse rate [26]. Above all, these findings provided further insights into the molecular mechanisms underlying this lymphoma, contributing to a more comprehensive understanding of TPM3‐ALK's role in tumorigenesis.

### 3.2 IMT

Clinically, IMT cases benefit from routine molecular confirmation (e.g., ALK testing) to identify patients eligible for ALK inhibitors; resistance to first‐generation TKIs can occur and should be anticipated in management. Notably, TPM3‐ALK is enriched in aggressive epithelioid inflammatory myofibroblastic sarcoma, underscoring its prognostic and therapeutic relevance. Lawrence et al. [27] initially highlighted its oncogenic potential, demonstrating activity in both lymphoid malignancies and mesenchymal neoplasms. Building on this, Hisaoka et al. provided direct evidence by identifying TPM3‐ALK fusion in IMTs. Importantly, they observed that ALK‐positive cells often lacked typical myofibroblastic markers [28]. Confirming these findings through an alternative approach, Elenitoba‐Johnson et al. [29] utilized proteomic analysis to detect the TPM3‐ALK fusion protein, reinforcing its presence and transformative role. Extending the scope, Kinoshita et al. [10] confirmed TPM3‐ALK in pulmonary IMTs and highlighted its potential as a reliable molecular marker for aiding clinical diagnostics. Mansfield et al. [30] emphasized this in a complex IMT case, stressing the necessity of molecular diagnostics for identifying fusion‐positive patients eligible for ALK inhibitors and noting challenges like crizotinib resistance. Further solidifying the central role of TPM3‐ALK, Lee et al. [31] linked it specifically to aggressive IMT subtypes like epithelioid inflammatory myofibroblastic sarcoma, demonstrating its contribution to constitutive ALK activation and tumor progression. In conclusion, the collective evidence robustly affirms the oncogenic role of TPM3‐ALK in IMTs, establishing it as a prime therapeutic target central to tumorigenesis and progression.

### 3.3 Renal Cell Carcinoma

Cajaiba et al. investigated ALK‐rearranged RCC in pediatric patients, identifying the TPM3‐ALK fusion in several cases and describing their distinct clinicopathological features. Their work suggests that characterizing the molecular profile of such RCCs is crucial for developing tailored therapeutic strategies [32]. Extending these findings beyond pediatrics, Thorner et al. reported a case of adult RCC harboring the TPM3‐ALK translocation, reinforcing its role across age groups. Importantly, this case occurred in a tumor exhibiting transcription factor E3 (TFE3) immunopositivity without the canonical TFE3 gene translocation, highlighting a specific diagnostic challenge [33]. Thorner et al. thus emphasized the critical need to explore alternative genetic alterations like TPM3‐ALK in such diagnostically ambiguous RCCs. Seo et al. discussed ALK‐rearranged RCC as a distinct subtype, noting that the TPM3‐ALK fusion is frequently identified among these cases in adolescents and adults (who typically lack sickle cell trait). This observation positions TPM3‐ALK as a defining genetic signature of this rare RCC subtype [34]. Yu et al. [35] further validated the presence of TPM3‐ALK fusions in RCC and significantly advanced the field by demonstrating that ALK inhibitors show promising efficacy against ALK‐rearranged RCC in their study, opening new avenues for targeted therapies. Echoing the diagnostic complexities noted by Thorner et al., Galea et al. detailed a case of ALK‐rearranged RCC with TPM3‐ALK fusion, emphasizing the challenges posed by its heterogeneous morphology. Their report strongly advocates for the critical role of confirmatory molecular testing (e.g., ALK‐FISH) in such cases, not only for accurate diagnosis but also to guide treatment selection including ALK inhibitors [36].

Collectively, these reports define a distinct subset of ALK‐rearranged RCC driven by TPM3‐ALK and provide a rationale for ALK inhibitor therapy.

### 3.4 Other Tumors

Recent studies have identified and begun to elucidate the role of the TPM3‐ALK fusion gene in various rare tumor types beyond its well‐established associations, shedding light on its significance in diverse tumorigenesis contexts and its emerging potential as a diagnostic marker. Yuan et al. described two cases of Spitz melanocytoma harboring the TPM3‐ALK fusion, representing one of the first reports of this fusion in this specific tumor type. Their detailed clinicopathological and molecular genetic analysis highlighted TPM3‐ALK's contribution to the development and behavior of these lesions. The study emphasizes the rarity of this fusion in Spitz melanocytomas, underscoring its importance as a novel and significant molecular finding [37]. While morphologically distinct, the presence of this kinase fusion can pose a diagnostic challenge, necessitating molecular confirmation to distinguish it from other ALK‐rearranged melanocytic tumors and to identify patients who may benefit from ALK inhibitor therapy, especially in cases with atypical features or local aggressiveness. In a similar vein, Wu et al. identified the TPM3‐ALK fusion among other fusion events in their analysis of spitzoid tumors. Their work demonstrated that TPM3‐ALK is present in a subset of spitzoid melanocytic neoplasms, illustrating how kinase fusions can drive tumorigenesis in these biologically indeterminate tumors and contribute to a deeper understanding of their molecular underpinnings. Significantly broadening the spectrum of TPM3‐ALK‐associated neoplasms, Linos et al. identified this fusion in a distinct entity—a cutaneous epithelioid vascular tumor. Their research suggests that TPM3‐ALK could play a crucial role in the pathogenesis of this type of vascular tumor, demonstrating the fusion's oncogenic potential beyond mesenchymal and melanocytic lineages [38]. This discovery enhances our understanding of molecular pathways in vascular tumors and underscores the power of genetic analysis to uncover novel oncogenic mechanisms in rare cancers [39].

In summary, these studies collectively demonstrate the expanding role of TPM3‐ALK as a significant oncogenic driver across a remarkably diverse range of rare tumors including melanocytic (Spitz melanocytoma, spitzoid neoplasms) and now vascular (cutaneous epithelioid vascular tumor) entities. The identification of this fusion gene provides crucial molecular markers for diagnosing these distinct subtypes and strongly suggests potential therapeutic avenues through ALK inhibitors.

## 4 TPM3‐NTRK1 Fusion Positive Tumors

The TPM3‐NTRK1 fusion gene has emerged as a significant oncogenic driver across various cancer types. This fusion leads to ligand‐independent activation of TRKA, promoting tumorigenesis via downstream signaling pathways such as MAPK/ERK and PI3K/AKT. Its discovery has revolutionized molecular oncology, allowing for targeted therapies using TRK inhibitors like larotrectinib and entrectinib. The discovery of NTRK gene fusions, particularly TPM3‐NTRK1, has highlighted the importance of molecular diagnostics in precision oncology. TPM3‐NTRK1 fusion proteins drive oncogenesis through constitutive activation of TRKA, resulting in downstream signaling cascade dysregulation. While rare, this fusion is present in multiple cancer types, often as a defining genetic alteration. The advent of TRK inhibitors has provided a histology‐agnostic treatment approach, yet challenges such as resistance mutations necessitate further exploration. Vaishnavi et al. summarized the role of NTRK1, NTRK2, and NTRK3 gene rearrangements in different tumor types. It revisits the TPM3‐NTRK1 fusion as an important oncogene, which has been one of the first identified transforming chromosomal rearrangements. The review highlights the growing list of tumors associated with TPM3‐NTRK1 including CRC, lung cancer, and thyroid cancer, and discusses its potential for targeted therapies [40]. Fuse et al. discussed resistance mechanisms in NTRK1‐rearranged cancers including TPM3‐NTRK1. They found that mutations such as G595R and G667C in NTRK1 contribute to resistance to NTRK inhibitors like entrectinib, highlighting the role of TPM3‐NTRK1 in these processe [41]. Kita et al. examined brain metastases models with TPM3‐NTRK1 fusions. They studied the effectiveness of entrectinib and crizotinib in treating these metastases, which showed distinct responses in the brain compared to other metastases [42]. Zhao et al. [43] identified NTRK gene fusions in lung adenocarcinomas, including TPM3‐NTRK1, which confirmed that TPM3‐NTRK1 is a rare but potentially actionable fusion in lung cancer. Koehne et al. demonstrated the use of pan‐TRK immunohistochemistry (IHC) for detecting NTRK gene fusions, including TPM3‐NTRK1, across different tumor types. They identify TPM3‐NTRK1 fusions in select cases and stress the utility of confirmatory RNA‐based NGS for accurate diagnosis [44]. Stockley et al. presented a Canadian ring study aimed at optimizing the detection of NTRK gene fusions in clinical molecular diagnostics, involving 16 laboratories across Canada. The study evaluated multiple RNA‐based NGS panels for the identification of NTRK fusions, including TPM3‐NTRK1, in various cancer samples, including lung adenocarcinoma and papillary thyroid carcinoma (PTC). The results showed variability in fusion detection across different panels, with TPM3‐NTRK1 fusion being detected in several samples, but not always with the same reliability. Specifically, the AmpliSeq for Illumina Focus panel faced challenges in detecting TPM3‐NTRK1 due to bioinformatics issues, highlighting the complexities in detecting low‐level or rare fusions. The study also pointed out the need for further refinement in bioinformatics pipelines to improve fusion detection, especially for fusions like TPM3‐NTRK1 that may have lower variant levels. This emphasizes the importance of optimizing NGS platforms and bioinformatics tools for more accurate and comprehensive NTRK fusion detection in clinical diagnostics [45].

### 4.1 Colorectal Cancer

Ardini et al. explored the role of TPM3‐NTRK1 rearrangement in colorectal cancer (CRC). They discovered that the TPM3‐NTRK1 fusion makes tumors highly sensitive to TRKA kinase inhibitors such as NMS‐P626, and shows significant antitumor activity in mouse models. The study suggests that the TPM3‐NTRK1 rearrangement could be a potential new therapeutic target in CRC [18]. Créancier et al. [46] identified TPM3‐NTRK1 fusions as rare but recurrent events in colorectal cancer, linked to constitutive TRKA activation and oncogenesis, suggesting TRK inhibitors as therapeutic targets. Ukkola et al. reported TPM3‐NTRK1 in mismatch repair‐deficient colorectal cancer. These fusions were enriched in microsatellite instability‐high tumors, with implications for precision medicine [47]. Sartore‐Bianchi described the identification of a novel LMNA‐NTRK1 gene fusion in a metastatic CRC patient, which was linked to sensitivity to the pan‐TRK inhibitor, entrectinib. This genetic alteration involved a rearrangement on chromosome 1, producing a chimeric protein with oncogenic potential. The patient, previously refractory to standard therapies, showed a partial response to entrectinib treatment, with a significant reduction in liver metastases. This is the first clinical evidence of the therapeutic efficacy of TRKA inhibition in solid tumors, providing insight into a genomic‐driven approach for targeting CRCs with such genetic alterations. This discovery suggests potential clinical applications for identifying other patients who could benefit from TRK‐targeted therapies [48]. Kato et al. found resistance to TRK inhibitors in colorectal cancer cells with TPM3‐NTRK1 fusions, mediated by overexpression of HMGCS2 and the mevalonate pathway. Combining TRK inhibitors with statins partially overcame this resistance [49]. Gatalica et al. [50] found TPM3‐NTRK1 fusions in colorectal cancer as a rare oncogenic driver, associated with high response rates to TRK inhibitors. Sohn et al. evaluated the effects of various TRK inhibitors on the TPM3‐NTRK1 fusion KM12SM colon cancer cell line. The study showed that TRK inhibitors induced apoptosis, inhibited the expression of NFκB and COX‐2, and enhanced the antioxidant NRF2 signaling pathway. Additionally, these drugs significantly inhibited the migration of KM12SM cells, suggesting that TRK inhibitors may suppress cancer progression by negatively regulating the NFκB pathway and positively regulating the NRF2 pathway [51]. Yamashiro et al. reported NTRK fusions in CRC and highlighted TPM3‐NTRK1 as one of the fusions identified. They noted the rarity of NTRK fusions in CRC, but emphasized their clinical relevance, as NTRK fusion‐positive tumors show sensitivity to TRK inhibitors [52]. Yonemaru et al. investigated NTRK fusions in CRC and reported that TPM3‐NTRK1 was present in a small subset of cases. The research found that these fusion‐positive tumors could benefit from TRK‐targeted therapies [53]. Kim et al. focused on the role of NTRK fusions in colorectal cancer, particularly in tumors that develop through the serrated neoplasia pathway. The study identified TPM3‐NTRK1 among other fusions in sessile serrated lesions [54]. Cho et al. focused on the anti‐tumor activity of AZD4547, a potent fibroblast growth factor receptor inhibitor, which was shown to inhibit TPM3‐NTRK1 fusion in colon cancer cells (KM12(Luc)). AZD4547 demonstrated significant anti‐proliferative effects, with a 50% growth inhibition of 49.74 nM against KM12(Luc), comparable to LOXO101, a known TRK inhibitor. The research suggests that AZD4547 could be a promising treatment for TPM3‐NTRK1 fusion‐driven cancers including those harboring the TPM3‐NTRK1 fusion [55]. Zhang et al. explored pan‐TRK IHC as a screening method for NTRK fusions in colorectal cancer. It found TPM3‐NTRK1 fusion in a subset of mismatch repair‐deficient colorectal tumors [56]. Wu et al. reported on colorectal cancer with NTRK rearrangements including TPM3‐NTRK1. The article focuses on the identification of NTRK fusions in colorectal cancer, particularly in mismatch repair‐deficient cases. It discusses the molecular characteristics of NTRK fusion‐positive CRCs, highlighting various fusion variants such as TPM3‐NTRK1. The study found that NTRK fusions were more prevalent in hypermethylated MLH1 CRCs, and detailed methods like IHC, FISH, and next‐generation sequencing (NGS) were used to detect these fusions. The TPM3‐NTRK1 fusion was identified in several cases, and the study noted that it displayed distinct immunohistochemical patterns including membrane and cytoplasmic positivity. This variant was compared to other NTRK fusions such as EML4‐NTRK3 and LMNA‐NTRK1, with varying FISH patterns observed, particularly in the subcellular localization of the fusion proteins. The findings underscore the importance of comprehensive testing for NTRK fusions to effectively identify targetable alterations in CRC [57]. Lasota et al. focused on colonic adenocarcinomas harboring NTRK fusion genes including TPM3‐NTRK1. The study discussed the various NTRK fusion variants identified such as LMNA‐NTRK1, TPR‐NTRK1, and EML4‐NTRK3, and highlights their clinical implications, particularly the potential for targeted therapies using Trk inhibitors [58].

### 4.2 Thyroid Carcinoma

Beimfohr et al. investigated NTRK1 rearrangements in childhood papillary thyroid carcinomas (PTCs) arising after the Chernobyl nuclear accident. Significantly, they identified TPM3‐NTRK1 as one of the rearrangements present in these radiation‐associated tumors, providing crucial molecular insights into the pathogenesis of this specific etiology of thyroid cancer [59]. Chu et al. characterized PTCs harboring TPM3‐NTRK1 fusions, linking these alterations to constitutive activation of the MAPK and PI3K/AKT pathways—key oncogenic drivers in thyroid cancer. Critically, their work provided clinical evidence demonstrating the efficacy of TRK inhibitors in patients with refractory PTCs positive for such fusions [12]. Pekova et al. conducted a broader analysis of NTRK fusions in thyroid cancer, confirming that TPM3‐NTRK1 represents a significant fusion type within a subset of PTCs. Importantly, their work detailed the distinct clinicopathological features commonly associated with NTRK fusion‐positive thyroid tumors and evaluated their significant impact on prognosis [60]. Zhang et al. [8] further supported these findings, specifically confirming TPM3‐NTRK1 in PTC and discussing its implications, particularly for the potential use of TRK inhibitors in advanced disease. Building upon the evidence of TRK inhibitor efficacy, Papadopoulos et al. [61] reported compelling direct clinical evidence from a Phase I trial: a patient with TPM3‐NTRK1 rearranged differentiated thyroid cancer treated with the NTRK/ROS1 inhibitor taletrectinib (DS‐6051b) achieved a durable partial response lasting 27 months, solidifying the targetability of this fusion.

### 4.3 Soft Tissue and Mesenchymal Tumors

The TPM3‐NTRK1 fusion gene has been increasingly recognized as a driver alteration in diverse soft tissue and mesenchymal tumors, with significant implications for diagnosis and targeted therapy. Evidence supporting TRK inhibitor efficacy is mounting, exemplified by reports such as Baranov et al. [62], who documented objective responses (including decreased cellularity and stromal hyalinization) to larotrectinib in pediatric mesenchymal tumors harboring TPM3‐NTRK1 fusions, and Huang et al. [63], who observed marked tumor shrinkage and necrosis in a pelvic spindle cell sarcoma patient with this fusion treated with larotrectinib. Diagnostically, the presence of the TPM3‐NTRK1 fusion defines distinct molecular subsets within various anatomical locations. Atiq et al. [64] described its occurrence in morphologically heterogeneous gastrointestinal mesenchymal tumors distinct from gastrointestinal stromal tumor. Kwon et al. identified TPM3‐NTRK1 in rare GIST‐like cases, while Gao et al. reported NTRK‐rearranged spindle cell neoplasms (including TPM3‐NTRK1) arising in the GI tract, noting their variable histological grades but responsiveness to TRK inhibitors [9, 65]. Critically, Croce et al. [66] revealed that a majority (7/13) of spindle cell sarcomas resembling fibrosarcoma in the uterus/vagina harbored NTRK rearrangements, primarily TPM3‐NTRK1 (6 cases), and crucially, all NTRK‐rearranged tumors exhibited diffuse Pan‐Trk immunohistochemical staining, highlighting a valuable diagnostic marker and defining a novel molecularly distinct entity. Yin et al. further confirmed the importance of NTRK rearrangements, including TPM3‐NTRK1, in pediatric soft tissue tumors, reinforcing the potential for targeted therapy in this population. Collectively, these findings underscore the critical role of molecular diagnostics (including Pan‐Trk IHC and fusion detection) in identifying TPM3‐NTRK1 fusion‐positive mesenchymal tumors across diverse sites and age groups and strongly support the therapeutic relevance of TRK inhibitors for this molecularly defined subset of sarcomas [67].

### 4.4 Cervical Sarcoma

Research by Chiang et al. [68] first identified TPM3‐NTRK1 fusions in cervical sarcoma, defining a novel fibrosarcoma‐like subtype responsive to TRK inhibitors. Nilforoushan et al. [69] subsequently emphasized the importance of detecting NTRK1 fusions in cervical sarcoma generally, highlighting their therapeutic vulnerability to TRK inhibitors. Supporting the relevance of NTRK fusions to cervical sarcomas specifically, Rabban et al. [70] reported three cases harboring these alterations (including TPM3‐NTRK1). Their study details distinct morphological and immunohistochemical features differentiating NTRK‐fusion cervical sarcomas from other uterine sarcoma types and underscores the positive response of these tumors to targeted therapies such as the TRK inhibitor larotrectinib. Collectively, these findings establish TPM3‐NTRK1 fusion as a defining molecular alteration in a novel, aggressive subtype of cervical sarcoma. Given the rarity and often dismal prognosis of uterine sarcomas, the identification of this fusion is of paramount diagnostic importance, as it delineates a distinct entity from its histological mimics and immediately opens avenues for targeted intervention with TRK inhibitors, representing a paradigm shift in the management of this disease.

### 4.5 NSCLC

Dong et al. found TPM3‐NTRK1 in lung adenocarcinoma (LADC), showing strong pan‐TRK positivity. TRK inhibitors proved effective in these rare cases [7]. Zhang et al. [8] reported TPM3‐NTRK1 in NSCLC, emphasizing the rarity of these fusions and their clinical importance in personalized medicine. Reischmann et al. identified mesenchymal‐epithelial transition factor (MET) amplification as a resistance mechanism in NTRK‐rearranged cancers including those with TPM3‐NTRK1 fusion. They emphasized the role of MET overexpression in NSCLC with NTRK1 mutations [71].

### 4.6 Glioma

Fang et al. identified TPM3‐NTRK1 in infant‐type hemispheric glioma. This high‐grade glioma responded to TRK inhibitors, expanding the genotypic spectrum of this entity [72]. Hardin et al. [73] reported NTRK fusions, including TPM3‐NTRK1, in pediatric low‐grade gliomas, demonstrating the clinical utility of RNA sequencing in diagnostics and therapy.

### 4.7 Other Tumors

The TPM3‐NTRK1 fusion gene emerges as a significant oncogenic driver across a spectrum of pediatric and other tumors, expanding the genetic landscape and offering critical therapeutic insights. Kao et al. [74] initially described its presence in fibrosarcoma‐like tumors in children, highlighting its potential role as a therapeutic target within this entity. This finding is reinforced by Gatalica et al. [50] who identified TPM3‐NTRK1 as a defining molecular feature in classic infantile fibrosarcoma and Agaram et al. [75] who characterized fusion‐positive cases within their series as infiltrative lipofibromatosis‐like neural tumors, directly implicating the TPM3‐NTRK1 fusion in their oncogenesis. Importantly, Huson et al. [73] demonstrated compelling clinical utility, reporting significant therapeutic benefit from TRK inhibitors in an infantile fibrosarcoma harboring TPM3‐NTRK1, even in the complex setting of Bloom syndrome, underscoring its potency as a target. The relevance of this fusion extends beyond fibrosarcomas. Wu et al. [38] identified recurrent TPM3‐NTRK1 fusions in morphologically challenging spitzoid melanomas and other biologically indeterminate tumors, indicating its contribution to the molecular heterogeneity of these lesions. Similarly, Acosta et al. [76] detected this fusion in tumors initially classified as prostatic stromal origin, further exemplifying its occurrence in diverse settings and the resulting diagnostic and biological complexity. The therapeutic implications, along with potential limitations, are sharply illustrated by Sun et al.'s report of a congenital mesoblastic nephroma (CMN) harboring TPM3‐NTRK1. This case documented an initial dramatic response to the TRK inhibitor larotrectinib, followed by the emergence of resistance, vividly highlighting both the promise and the clinical challenges associated with targeting this fusion [56].

## 5 TPM3‐ROS1 Fusion‐Positive Tumors

### 5.1 NSCLC

TPM3‐ROS1 fusion‐positive NSCLC has emerged as a compelling therapeutic target. Zhao et al. [82] first identified this oncogenic driver in approximately 4% of NSCLC patients using cell block samples. Notably, these patients exhibited significantly improved disease control rates and progression‐free survival following treatment with the TKIs crizotinib compared to fusion‐negative cohorts, underscoring TPM3‐ROS1's targetability. Further validation of its therapeutic relevance was demonstrated by Yokota et al. [77] using patient‐derived lung organoids harboring TPM3‐ROS1 fusions. Their work revealed marked tumor growth suppression upon exposure to both crizotinib and entrectinib, reinforcing the clinical rationale for TKIs‐based targeting.

Despite these promising responses, acquired resistance represents a significant therapeutic challenge. Tyler et al. [78] identified MET gene amplification as a mechanism conferring bypass resistance to entrectinib, suggesting that combinatorial targeting of MET and ROS1 may overcome this limitation. Additionally, Thawani et al. [79] characterized the ROS1 L2086F gatekeeper mutation in TPM3‐ROS1‐positive models, which confers resistance to first‐generation ROS1 inhibitors. This finding highlights the necessity for next‐generation inhibitors (e.g., cabozantinib) capable of overcoming mutation‐driven resistance.

Comprehensive molecular profiling is further warranted by reports of rare molecular complexities. Zhu et al. [14] documented a unique case of concomitant EML4‐ALK and TPM3‐ROS1 fusions within a single NSCLC tumor, illustrating the critical need for broad genomic screening. Similarly, Cai et al. [80] reported a TPM3‐ROS1‐positive metastatic SpCC in a 72‐year‐old patient, detected via NGS. This patient achieved sustained clinical benefit from crizotinib, with reduced tumor burden, resolution of pleural effusion, and improved quality of life over 13 months. Tan et al. [81] further emphasized the importance of multi‐lesion profiling in a case of synchronous bilateral lung adenocarcinomas, revealing spatially distinct drivers—EML4‐ALK in the right lobe and TPM3‐ROS1 in the left—confirmed as independent primaries by genomic analysis.

Collectively, these findings establish TPM3‐ROS1 fusion as a bona fide oncogenic driver in NSCLC and highlight the necessity for molecularly guided therapeutic strategies. While TKIs like crizotinib offer significant clinical benefit, emerging resistance mechanisms demand next‐generation inhibitors and rational combination therapies. Comprehensive genomic profiling remains indispensable for optimizing personalized treatment in rare, molecularly complex NSCLC subtypes.

## 6 Discussion

The intricate landscape of TPM3 gene fusions illuminates a paradigm shift in precision oncology, where structural genomic rearrangements transcend histopathological boundaries to define actionable therapeutic vulnerabilities. As this review synthesizes, TPM3—a cytoskeletal regulator critical for actin filament stabilization—becomes an oncogenic catalyst when fused to tyrosine kinase domains of NTRK1, ALK, or ROS1. The resulting chimeric proteins constitutively activate kinase signaling cascades (MAPK/ERK, PI3K/AKT, JAK/STAT), driving tumorigenesis across epithelial, mesenchymal, and neural malignancies (Table 1).

**TABLE 1:** TPM3‐associated signaling pathways, targeted therapies and reported resistance mutations and mechanisms.

Type of TPM3 gene fusions	Name of signaling pathways	Targeted therapies	Reported resistance mutations/mechanisms	References
TPM3‐ALK	JAK/STAT, RAS/MAPK	Crizotinib, ceritinib	Gatekeeper mutation	[13, 21]
TPM3‐NTRK1	MAPK/ERK, PI3K/AKT	Larotrectinib, entrectinib	NTRK1, G595R, G667C	[12, 18, 51]
TPM3‐ROS1	MAPK/ERK, PI3K/AKT	Crizotinib, entrectinib	ROS1, L2086F, MET amplification	[9, 80]

### 6.1 Molecular Convergence and Clinical Heterogeneity

Despite divergent fusion partners, TPM3 fusions share mechanistic hallmarks: coiled‐coil domain‐mediated dimerization, ligand‐independent kinase activation, and downstream pathway dysregulation. A key insight from this synthesis is that the common molecular architecture of these fusions, rather than the tissue of origin, dictates both the pathogenic mechanism and the therapeutic vulnerability. This molecular convergence underpins their “histology‐agnostic” targetability, exemplified by TRK inhibitors (larotrectinib/entrectinib) inducing responses in TPM3‐NTRK1+ colorectal carcinomas, gliomas, and sarcomas. Similarly, ALK/ROS1 inhibitors (crizotinib, ceritinib) demonstrate efficacy in inflammatory myofibroblastic tumors (IMTs), NSCLC, and renal cell carcinomas (RCCs) harboring TPM3‐ALK/ROS1 fusions. As illustrated in Figure 1 of this review, TPM3 gene fusions are distributed across a wide array of tumors with diverse histological origins. This broad distribution pattern underscores the necessity of screening for NTRK, ALK, and ROS1 fusions in cross‐cancer clinical practice, particularly for rare or refractory malignancies with limited standard treatment options. Nevertheless, tumor microenvironmental pressures foster heterogeneous resistance mechanisms. MET amplification bypasses ROS1 inhibition in NSCLC; NTRK1 solvent‐front mutations (G595R/G667C) compromise TRK inhibitor efficacy, and ALK gatekeeper mutations drive relapse in IMTs—highlighting the imperative for next‐generation inhibitors (e.g., cabozantinib, repotrectinib).

### 6.2 Diagnostic and Therapeutic Imperatives

The rarity of TPM3 fusions (< 4% in NSCLC, < 1% in CRC) demands robust molecular screening. RNA‐based NGS and pan‐TRK/ALK IHC are indispensable, yet technical challenges persist—as evidenced by variable TPM3‐NTRK1 detection in multi‐institutional ring studies. Crucially, comprehensive profiling uncovers co‐occurring drivers (e.g., synchronous EML4‐ALK and TPM3‐ROS1 in bilateral lung adenocarcinomas) or histologic mimics (e.g., TPM3‐ALK+ cutaneous epithelioid vascular tumors masquerading as melanocytic lesions). The complexity involved requires the implementation of tissue‐agnostic screening protocols that integrate DNA/RNA sequencing to capture the diversity of fusions. Additionally, lesion‐specific profiling is essential to address spatial heterogeneity in multifocal tumors. Furthermore, functional validation using patient‐derived organoids is necessary to prioritize targetable fusions.

### 6.3 Future Directions

While TKIs revolutionized outcomes, durable responses are hampered by intrinsic/acquired resistance. Combinatorial strategies—MET/ROS1 co‐inhibition, TRK/MEK blockade, or immunotherapy synergy—warrant exploration. Moreover, the ontogeny of TPM3 fusions in radiation‐associated malignancies (e.g., post‐Chernobyl thyroid carcinomas) suggests DNA repair defects as potential susceptibility biomarkers. Prospective registries (e.g., Low‐Grade Glioma in Children for pediatric gliomas) must correlate fusion variants with clinical trajectories to refine risk stratification.

## 7 Conclusion

The comprehensive analysis of TPM3 gene fusions presented in this review underscores their significant role as oncogenic drivers across a diverse spectrum of malignancies including inflammatory myofibroblastic tumors, anaplastic large cell lymphoma, renal cell carcinoma, colorectal cancer, thyroid carcinoma, non‐small cell lung cancer, soft tissue sarcomas, and glioma. These fusions constitutively activate receptor tyrosine kinase signaling pathways (MAPK/ERK, PI3K/AKT, JAK/STAT) through mechanisms involving TPM3 coiled‐coil domain‐mediated dimerization, thereby driving uncontrolled proliferation, survival, and metastasis. Critically, the identification of these fusions has profound therapeutic implications, as they represent actionable targets for specific TKIs (e.g., larotrectinib/entrectinib for NTRK1, crizotinib/ceritinib for ALK/ROS1), enabling histology‐agnostic treatment paradigms that have demonstrated significant clinical responses. However, the clinical benefit is often challenged by the emergence of resistance mechanisms and the complexities of tumor heterogeneity and spatial genomic diversity, exemplified by cases harboring concurrent fusions like EML4‐ALK and TPM3‐ROS1. Overcoming these challenges necessitates robust molecular diagnostics (leveraging RNA‐based NGS and validated pan‐TRK/ALK IHC), the development of next‐generation inhibitors capable of bypassing common resistance mutations, rational combinatorial strategies, and comprehensive lesion‐specific profiling to accurately capture the molecular landscape and guide effective, personalized therapeutic interventions for patients harboring these oncogenic drivers. By systematically cataloging and analyzing TPM3 fusions, this review provides a rationale for future clinical trial design and underscores the necessity of molecular profiling in precision oncology, thereby aiming to directly influence both research priorities and patient care.

## Author Contributions

**Anjie Chen:** writing – original draft, writing – review and editing, conceptualization, software. **Sixin Li:** writing – original draft, methodology, writing – review and editing. **Chen Guo:** writing – review and editing. **Chenwei Gu:** writing – review and editing. **Jiandong Gui:** data curation, writing – review and editing. **Yujie Deng:** data curation, writing – review and editing. **Xichen Feng:** visualization, writing – review and editing. **Yuanyuan Mi:** supervision, funding acquisition, writing – review and editing, project administration, conceptualization, resources.

## Funding

This work is supported by the National Natural Science Foundation (no. 81802576), the Wuxi Commission of Health and Family Planning (No. M202330), the Talent Plan of Taihu Lake in Wuxi (Double Hundred Medical Youth Professionals Program) from the Health Committee of Wuxi (No. BJ2023051), and Jiangsu Province 7th phase “333” high‐level talents.

## Ethics Statement

Review and/or approval by an ethics committee was not needed for this study because this is a review.

## Consent

The authors have nothing to report.

## Conflicts of Interest

The authors declare no conflicts of interest.

## Data Availability

Data sharing not applicable to this article as no datasets were generated or analysed during the current study.
